# NAFLD/MASLD and the Gut–Liver Axis: From Pathogenesis to Treatment Options

**DOI:** 10.3390/metabo14070366

**Published:** 2024-06-28

**Authors:** Natalia G. Vallianou, Dimitris Kounatidis, Sotiria Psallida, Nikolaos Vythoulkas-Biotis, Andreas Adamou, Tatiana Zachariadou, Sofia Kargioti, Irene Karampela, Maria Dalamaga

**Affiliations:** 1 First Department of Internal Medicine, Sismanogleio General Hospital, Sismanogliou 1 Str., 15126 Athens, Greece; 2 Department of Internal Medicine, Hippokration General Hospital, 114 Vassilissis Sofias Str., 11527 Athens, Greece; dimitriskounatidis82@outlook.com; 3 Department of Microbiology, “KAT” General Hospital of Attica, 14561 Athens, Greece; sotiriapsallida@gmail.com; 4 Second Department of Critical Care, Attikon University Hospital, 1 Rimini Str., 12462 Athens, Greece; eikaras1@gmail.com; 5 Department of Biological Chemistry, National and Kapodistrian University of Athens, 75 Mikras Asias Str., 11527 Athens, Greece; madalamaga@med.uoa.gr

**Keywords:** fatty liver, fecal microbiota transplantation, gut dysbiosis, gut microbiota, metabolic dysfunction-associated steatotic liver disease, nonalcoholic fatty liver disease, phage, prebiotics, probiotics, postbiotics

## Abstract

Nonalcoholic fatty liver disease (NAFLD) poses an emerging threat topublic health. Nonalcoholic steatohepatitis (NASH) is reported to be the most rapidly rising cause of hepatocellular carcinoma in the western world. Recently, a new term has been proposed: metabolic dysfunction-associated steatotic liver disease (MASLD). The introduction of this new terminology has sparked a debate about the interchangeability of these terms. The pathogenesis of NAFLD/MASLD is thought to be multifactorial, involving both genetic and environmental factors. Among these factors, alterations in gut microbiota and gut dysbiosis have recently garnered significant attention. In this context, this review will further discuss the gut–liver axis, which refers to the bidirectional interaction between the human gut microbiota and the liver. Additionally, the therapeutic potential of probiotics, particularly next-generation probiotics and genetically engineered bacteria, will be explored. Moreover, the role of prebiotics, synbiotics, postbiotics, and phages as well as fecal microbiota transplantation will be analyzed. Particularly for lean patients with NAFLD/MASLD, who have limited treatment options, approaches that modify the diversity and composition of the gut microbiota may hold promise. However, due to ongoing safety concerns with approaches that modulate gut microbiota, further large-scale studies are necessary to better assess their efficacy and safety in treating NAFLD/MASLD.

## 1. Introduction

Nonalcoholic fatty liver disease (NAFLD) refers to the abnormal accumulation of lipids in the liver that is not related to alcohol consumption. This hepatic fat accumulation results from excess fat in the human body which is deposited in the liver among other organs [1,2,3]. NAFLD may progress to chronic liver diseases with various degrees of inflammation, known as nonalcoholic steatohepatitis (NASH) and fibrosis, which may lead to cirrhosis and ultimately hepatocellular carcinoma [3,4,5]. NAFLD is a major public health problem affecting approximately 25% of the population worldwide [5,6,7]. In 2023, a Delphi consensus statement introduced the use of metabolic dysfunction-associated steatotic liver disease (MASLD) instead of NAFLD. This new nomenclature highlights the importance of the presence of liver steatosis and at least one out of five cardiometabolic risk factors: (1) body mass index (BMI) ≥ 25 kg/m^2^ for Caucasians (BMI ≥ 23 kg/m^2^ for Asians) or waist circumference (WC) > 90 cm (in males) and 80 cm (in females), classified as BMI subgroup; (2) fasting serum glucose ≥ 5.6 mmol/L, 2 h post-load glucose levels ≥ 7.8 mmol/L, HbA1c ≥ 5.7%, diagnosis of diabetes, or treatment for diabetes, classified as diabetes subgroup; (3) blood pressure ≥ 130/85 mmHg or specific antihypertension treatment, classified as hypertension subgroup; (4) plasma triglycerides ≥ 1.70 mmol/L or lipid-lowering treatment, defined as triglyceride subgroup; (5) plasma high-density lipoprotein cholesterol(HDL) ≤ 1.0 in males and ≤1.3 mmol/L in females or lipid-lowering treatment, classified as HDL subgroup. Patients with hepatic steatosis who do not meet the cardiometabolic risk factors are diagnosed as cryptogenic SLD. These five cardiometabolic risk factors are well-known factors in establishing the diagnosis of metabolic syndrome (MS), as reported by the International Diabetes Federation (IDF). In this context, metabolic-associated steatohepatitis (MASH) has been substituted for NASH [8,9,10]. The introduction of this new terminology has sparked a debate about the interchangeability of NAFLD/NASH and MASLD/MASH. Nowadays, NASH/MASH is suggested to be the most rapidly rising cause of hepatocellular carcinoma and the most frequent cause of liver transplantation among females in the United States of America [11,12,13].

The human microbiota constitute the sum of microorganisms inhabiting the human body. Although this sum of microorganisms extends beyond the gut and is distributed throughout the human body, we commonly refer to it as the “gut microbiota” since the majority of microorganisms in the human body inhabit the gut [14,15,16]. The human microbiome comprises the sum of each and every gene from the bacteria, archaea, viruses, and eukaryotic microbes that inhabit the human body. In adults, the gut bacteria belong mainly to two phyla, the Gram-positive *Firmicutes* and the Gram-negative *Bacteroidetes* [14,15,16]. Under normal circumstances, there exists a state of equilibrium between the gut microbiota and the host. However, under the influence of various genetic and environmental factors, an imbalance between the gut microbiota and the host occurs, known as “gut dysbiosis”. Genome-wide association studies (GWAS) have associated NAFLD with single nucleotide polymorphisms (SNPs), mainly in the *PNPLA3* (patatin-like phospholipase domain containing 3), the *TM6SF2* (transmembrane 6 superfamily member 2), the *MBOAT7* (membrane bound O acyltransferase 7), and the *GCKR* (glucokinase regulator) [17]. Regarding environmental components, the most widely accepted to promote NAFLD/MASLD are the Western diet, the use of antibiotics, and a sedentary lifestyle [18]. Gut dysbiosis has been proposed to play a role in the pathogenesis of NAFLD/MASLD through the gut–liver axis. The objective of this review is to summarize recent data on the role of the gut–liver axis in the development of NAFLD/MASLD. Furthermore, in addition to exploring the pathogenetic pathways of this disease, we will strive to clarify the potential utility of probiotics, prebiotics, and synbiotics in managing NAFLD/MASLD. In this regard, the alteration of gut microbiota through the use of probiotics, genetically engineered bacteria, prebiotics, synbiotics, postbiotics, phages, and fecal microbiota transplantation (FMT) will be further examined.

## 2. Pathogenesis of NAFLD/MASLD: The Gut-Liver Axis

The gut–liver axis is a term used to describe the complex interplay between the gut epithelial, vascular, and immunological barriers and the liver circulation in the context of the gut microbiota’s composition and functionality [19,20,21,22,23]. The gut barrier consists of a mucus layer, with an outer thinner layer and an inner thicker layer, after which lie the epithelial cells that serve as the second main barrier. Epithelial cells are tied together with tight junctions (TJs). TJs are proteins, including mainly claudins, occludin, and zonula occludens-1 protein as well as junctional adhesion molecules (JAMs) [23,24,25,26,27]. TJs play a crucial role in maintaining the integrity of the gut epithelial barrier. By their structure and function, they impede the invasion of the intestinal epithelial cells (IECs) by microbial pathogens, while allowing for the entry of various nutrients [28,29]. Apart from the mucin layer produced by goblet cells and the TJs between the IECs, equally important is the involvement of the immune system. More specifically, IgA, secreted by plasma cells locally, binds and neutralizes invading microorganisms. In addition, the release of interleukin-23 (IL-23) induces the activation of group 3 innate lymphoid cells, which, in turn, produce IL-22. IL-22 seems to mediate the production of antimicrobial peptides from Paneth cells as well as from IECs. Furthermore, vascular and lymphoid barriers are also implicated in the whole process. In particular, most of the small and large intestinal blood flow ends at the portal vein level, thereby reaching the liver sinusoids. In this way, the endothelial sinusoidal cells activate the Kupffer cells, which translocate into the periportal area to further defend the host from pathogens and gut-derived toxins, such as trimethylamine (TMA), p-cresol (PC), and H_2_S [28,29,30]. Notably, the liver contains fewer T cells, which are also less proficient at defending against invaders compared to those in the intestines. This gut–liver defending mechanism is bidirectional, i.e., there is a liver-to-gut component as well. This component comprises bile, which mainly consists of bile acids (BAs), IgA, antimicrobial peptides, and bicarbonates. Its mixture exhibits profound host-defending features. For example, BAs exert antibacterial potential directly, due to their detergent properties, and indirectly, by activating the farnesoid X receptor (FXR). More specifically, the nuclear FXR is the receptor of BAs. BAs are classified as primary, such as cholic acid and chenodeoxycholic acid, and secondary BAs. Primary BAs may conjugate with glycine or taurine by the hepatocytes before being secreted in the bile by the bile salt export pump (BSEP). Secondary BAs are formed in the intestines by de-conjugation by the gut microbiota [31,32]. Bacteria such as *Bacteroidetes*, *Lactobacillus*, *Bifidobacterium*, and *Clostridium* are suggested to be involved in this de-conjugation process by producing bile salt hydrolase (BSH) [32,33]. BAs serve as the endogenous ligand of FXR, with chenodeoxycholic acid being the most abundantly bound to FXR. The stimulation of FXR by BAs, in addition to self-regulating the composition of BAs, can lead to the modulation of several transcription factors associated with lipogenesis, inflammation, and fibrosis, all of which are well-known inherent features of NAFLD/MASLD [31,32,33,34]. FXR is also implicated in alterations in the permeability of the intestinal epithelial barrier, thus playing a pivotal role in shaping the gut microbiota. Notably, the activation of the FXR results in the secretion of antimicrobial peptides by IECs. FXR may also be activated in the ileum in the FXR/FGF15/FGF19 pathway (fibroblast growth factor 15 in rodents or fibroblast growth factor 19 in humans) [34,35]. Therefore, the activation of FXR by FXR analogues that could prevent de novo lipogenesis, inflammation, and fibrosis may be beneficial in ameliorating NAFLD/MASLD [31,32,33,34,35].

Through the gut–liver axis, a critical defense mechanism is established, aiming to confine and eliminate invading pathogens and toxins to prevent systemic inflammation. However, this function is only effective under normal conditions. Conversely, when gut dysbiosis occurs, this multi-target defensive mechanism malfunctions. Regarding NAFLD/MASLD, gut dysbiosis is characterized by alterations in the composition and diversity of the gut microbiota [11,12]. In particular, most studies have revealed that patients with NAFLD/MALSD have an increased abundance of the Gram-negative *Bacteroidetes*, resulting in a decreased *Firmicutes* to *Bacteroidetes* (*F*/*B*) ratio. When compared to healthy controls, patients with NAFLD/MASLD exhibit an enhancement in *Enterobacterales* and *Proteobacteria*, whereas they have a decreased abundance of *Akkermansiamuciniphila* (*A. muciniphila*) and *Faecalibacteriumprausnitzii* (*F. prausnitzii*) [36]. This phenomenon could be attributed to the reduction in TJs that has been documented among mice fed a high-fat diet (HFD). Specifically, TJs protect from the invasion of pathogen-associated molecular patterns (PAMPs) in the gut. Indeed, in animal models of NAFLD/MASLD, there is a significant decrease in the number of TJs when mice are fed a HFD. This reduction in TJs could account for the increased permeability of PAMPs and the so-called “leaky gut” [37,38]. Therefore, promoting TJ’s function could restore the intestinal barrier’s integrity. In addition, toll-like receptors (TLRs), mainly TLR4 and TLR9, have been suggested to play a crucial role in the inflammatory process that drives NASH/MASH. The lipopolysaccharide (LPS) of Gram-negative bacteria passes through the leaky gut to the portal vein and activates TLRs. In turn, this TLR activation results in the activation of NF-*k*B as well as the inflammasome NLRP3, leading to various degrees of hepatic inflammation via the secretion of pro-inflammatory cytokines, as seen in NASH/MASH [39,40].

Concerning NAFLD/MASLD and variations in the composition and diversity of the gut microbiota, these alterations vary between patients with NAFLD/MASLD and obesity in comparison to lean patients with NAFLD [11,12]. Lee et al. documented that patients in Asia with NAFLD/MASLD and obesity had differential microbial signatures, when compared to non-obese Asian patients with NAFLD. More specifically, patients in Asia with NAFLD/MASLD and obesity had lower levels of *Ruminococcaceae* and an increased abundance of *Veillonellaceae*, and exhibited low diversity in their gut microbiota as well. The aforementioned changes were also associated with fibrosis severity levels [41]. Even the gut virome and the gut mycobiome are different among patients with NAFLD/MASLD [42,43,44,45]. Lang et al. have documented that patients with NAFLD/MASLD and an increased NAS (NAFLD activity score) exhibited decreased bacteriophage diversity in their fecal virome when compared to patients with NAFLD/MASLD and a low NAS [43]. Moreover, Demir et al. have shown that non-obese patients with more severe forms of NAFLD/MASLD had a distinct fecal mycobiome when compared to obese patients with less severe NAFLD/MASLD [44].

**Figure 1:**
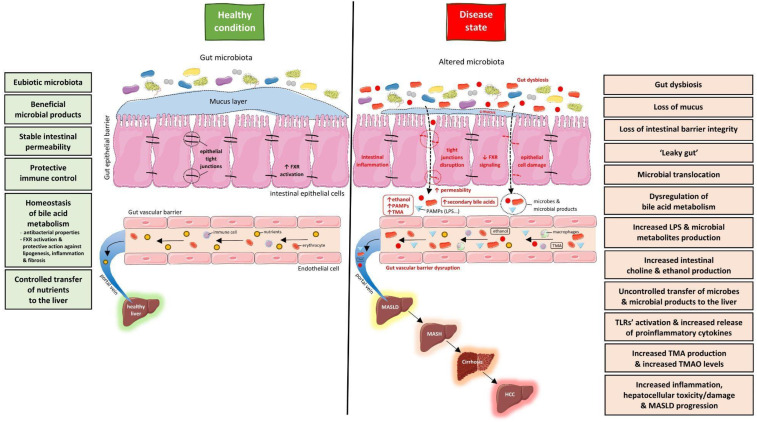
Gut–liver axis under normal circumstances and when gut dysbiosis occurs, and its connection with NAFLD/MASLD progression and pathogenesis. Abbreviations: FXR, farnesoid X receptor; HCC, hepatocellular carcinoma; LPS, lipopolysaccharide; MASLD, metabolic dysfunction-associated steatotic liver disease; MASH, metabolic dysfunction-associated steatohepatitis; PAMPs, pathogen associated molecular patterns; TLRs, toll-like receptors; TMA, trimethylamine; TMAO, trimethyl-N-amine oxide (parts of the figure originated from the free medical site http://smart.servier.com/ (accessed on 15 March 2024) by Servier licensed under a Creative Commons BY 4.0 License https://creativecommons.org/licenses/by/4.0/ (accessed on 15 March 2024)).

It is also noteworthy that, among patients with NAFLD/MASLD, gut dysbiosis is characterized by an increased production of TMA by the gut microbiota. The dietary precursors of TMA are mainly choline, phosphatidylcholine, betaine, and L–carnitine, which are abundant in eggs, red meat, and fish [46]. Significantly, although the gut microbiota play a pivotal role in the production of TMA, only a small fraction of gut microbes have the capability to convert its dietary precursors into TMA [46]. After its production in the gut, TMA, via the enterohepatic circulation, is transformed into trimethyl-N-amine oxide (TMAO) in the liver by monooxygenases. TMAO has been proposed as a risk factor in the development of NAFLD/MASLD [46]. Furthermore, increased serum levels of TMAO have been associated with the severity of liver steatosis among patients with NAFLD/MASLD [47]. Apart from TMA and LPS, hyperammonemia has also been associated with NAFLD/MASLD. In particular, ammonia is produced by the fermentation of proteins by gut bacteria. This process results in an increased production of ammonia and branched-chain fatty acids (BCFAs). Consequently, hyperammonemia and increased levels of BCFAs are believed to play a role in the development and progression of NAFLD/MASLD [48]. Figure 1 illustrates the principal components of the gut–liver axis and their interaction under normal conditions, as well as when gut dysbiosis occurs, in relation to the pathogenesis of NAFLD/MASLD.

### 2.1. The Potential Role of Probiotics and Genetically Engineered Microbes in the Treatment of NAFLD/MASLD

Probiotics, defined as “live microorganisms that confer a health benefit when consumed in adequate amounts”, as proposed by the World Health Organization (WHO) in 2002, have been increasingly studied lately [49]. The concept of altering the gut microbiota through the administration of live microorganisms that provide beneficial effects, though simple, appears promising. Regarding NAFLD/MASLD and probiotics, most studies, until today, have been performed in animal models. Table 1 comprises the main studies in animal models regarding probiotics administration and NAFLD parameters during the past five years. These studies have mainly used *Lactobacillus* or *Bifidobacterium* species or mixtures of these probiotics [11,12]. Readily prepared mixtures of probiotics, such as VSL#3, seem to be more promising than solely single probiotics. VSL#3 is a high-concentration probiotic mixture of eight probiotics, consisting of one strain of *Streptococcus thermophilus* BT01, three strains of *Bifidobacterium* (*B. breve* BB02, *B. animalis* subspecies *lactis* BL03, and *B. animalis* subsp. *lactis* BI04), and four strains of *Lactobacillus* (*L. acidophilus* BA05, *L. plantarum* BP06, *L. paracasei* BP07, and *L. helveticus* BD08) [50]. Aside from the research conducted in animal models, Denosa et al. [51] have administered VSL#3 for 12 weeks in adult patients with NAFLD/MASLD. In their randomized clinical trial, Denosa et al. enrolled 60 patients with NAFLD/MASLD and showed that the administration of two sachets of VSL#3 improved serum triglyceride levels and inflammatory markers, such as hs-CRP (high-sensitivity C-reactive protein). In addition, supplementation with VSL#3 resulted in reductions in serum gamma-glutamyltransferase levels, transaminase levels, and the hepatic steatosis index [51]. Nevertheless, in their randomized, double-blinded study, Chong et al. have shown that the administration of VSL#3 daily for 10 weeks in patients with NAFLD/MASLD did not ameliorate liver function. However, there was an improvement in inflammatory markers, such as hs-CRP, as well as in the insulin resistance index (HOMA-IR) [52]. Ahn et al. have administered another probiotics mixture containing six probiotics, namely *Lactobacillus acidophilus*, *L. rhamnosus*, *L. paracasei*, *Pediococcuspentosaceus*, *Bifidobacterium lactis*, and *B. breve* [53]. In their randomized, double-blinded study, Ahn et al. recruited 68 obese patients diagnosed with NAFLD/MASLD. They observed a noteworthy decrease in both body weight and intrahepatic fat, evaluated through magnetic resonance imaging (MRI) and the proton density fat fraction (PDFF), following a 12-week supplementation with the probiotics mixture [53]. Moreover, Duseja et al. [54] have demonstrated an amelioration in the histological findings in liver biopsies of 19 patients with NAFLD/MASLD, after the administration of a 16-strain probiotics mixture for one year, when compared to 20 patients who received placebo. More specifically, they showed a reduction in hepatocellular ballooning and fibrosis with an improvement in serum alanine transferase levels (ALT), tumor necrosis factor alpha (TNF-a), serum leptin, and endotoxin levels. Therefore, they concluded that this mixture resulted in an improvement in major histological parameters of NAFLD/MASLD as well as in decreases in cytokine levels and serum ALT [54]. However, there are many studies which do not support a beneficial effect of probiotics mixture supplementation among patients with NAFLD/MASLD. For example, in their randomized, double-blinded, placebo-controlled study of 39 patients with NAFLD/MASLD, Nor et al. evaluated the administration of a probiotics mixture containing six strains of *Lactobacillus* and *Bifidobacterium* (MCP^®^ BCMC^®^ strains). Nor et al. did not find any significant improvement regarding liver steatosis and serum liver indices after supplementation with MCP^®^ BCMC^®^ strains for 6 months [55]. In addition, Silva-Sperb et al. only recently reported the results of the PROBILIVER clinical trial, regarding the administration of a probiotics mixture consisting of *Lactobacillus acidophilus*, *Lactobacillus rhamnosus*, *Lactobacillus paracasei*, and *Bifidobacterium lactis* for 24 weeks. Among the 44 adult patients with biopsy-proven NASH/MASH, they did not find any improvement regarding liver function, as assessed by serum liver enzymes, transient elastography, NAFLD fibrosis score, and fatty liver index calculations [56]. Overall, there are inconclusive results regarding the administration of probiotics, even in the early stages of NAFLD/MASLD [55,56].

Nevertheless, the therapeutic potential of relatively novel probiotics, the so-called “next generation probiotics”, is currently being evaluated. Nowadays, *A. muciniphila* and *F. prausnitzii* are the major next generation probiotics being studied. The special interest in these probiotics has stemmed from the results of several studies supporting a decreased abundance of these two bacteria in the gut microbiome in NAFLD/MASLD [84,85]. *A. muciniphila* is a Gram-negative, strictly anaerobe, mucin-degrading bacterium which promotes the production of short chain fatty acids (SCFAs). SCFAs serve as energy sources for IECs and concurrently exhibit immunomodulatory properties within the gut. In particular, *A. muciniphila* has been documented to improve the function of TJs in the intestinal epithelial barrier [86]. In addition, the outer membrane of *A. muciniphila* protein Amuc-1100, which remains stable after pasteurization, seems to exert beneficial effects, such as stimulation of the secretion of glucagon-like peptide-1 (GLP-1), while facilitating lipolysis and decreasing the gut barrier’s permeability. GLP-1 is an incretin derived from enteroendocrine L-cells, which are more abundant in the distal ileum, but may be found in the jejunum and the duodenum [87]. GLP-1 reduces serum glucose levels by increasing the secretion of insulin. Furthermore, GLP-1 inhibits gastric emptying and thus induces satiety. The secretion of GLP-1 in the central nervous system (CNS) partly accounts for the delay in gastric emptying and the decreased appetite. Moreover, Ottman et al. have reported that Amuc-1100, by interacting with TLR-2 and TLR-4, may result in an increased production of the anti-inflammatory cytokine IL-10 [88]. Besides, *A. muciniphila* has been documented to be involved in controlling the polarization of macrophages in HFD-induced animal models of NAFLD. More specifically, *A. muciniphila* has been shown to reduce liver pro-inflammatory M1 macrophages as well as γδT and γδT17 cells in NASH/MASH [89]. The collective metabolic and anti-inflammatory effects are proposed to contribute to the therapeutic potential of *A. muciniphila* in NAFLD/MASLD. Very recently, in 2024, Wu et al. reported upon the effects of supplementation with *A. muciniphila* and VSL#3 in a MCD mice model of NAFLD. They concluded that both probiotics ameliorated NASH parameters, but *A. muciniphila* exhibited greater effectiveness in reducing liver fat accumulation, while VSL#3 demonstrated superiority in reducing intestinal barrier permeability and inflammation [89]. However, it should be noted that there is a considerable dearth of studies among humans with NAFLD/MASLD and supplementation with this next-generation probiotic.

*F. prausnitzii* is a Gram-positive, anaerobic bacterium which may produce SCFAs in the gut [90]. Its abundance is decreased in the gut in the context of NAFLD/MASLD [90]. Hu et al. have recently assessed the efficacy of two strains of this next-generation probiotic, namely LC49 and LB8, in a mouse model of NAFLD. Hu et al. have confirmed the positive association between the administration of *F. prausnitzii* LC49 and LB8 and improvement in NAFLD. They attributed this improvement to alterations in the gut microbiota as well as to changes in metabolic pathways [91]. *F. prausnitzii* has been a key player in maintaining gut homeostasis, mainly by producing SCFAs, especially butyrate. As already aforementioned, SCFAs help restore gut dysbiosis by promoting the intestinal barrier’s integrity and by exerting anti-inflammatory properties. More specifically, *F. prausnitzii* may inhibit NF-*k*B (nuclear factor kappa-light-chain-enhancer of activated B cells) and increase the levels of anti-inflammatory cytokines, such as IL-10, while decreasing pro-inflammatory cytokines, such as IL-6 and TNF-a [92]. Overall, while next-generation probiotics show promise for treating NAFLD/MASLD, it is essential to emphasize the need for more robust data and additional research involving patients with NAFLD/MASLD [91,92].

Recently, efforts have been directed towards genetically modifying probiotics, recognizing that the effectiveness of probiotics may be compromised by issues related to absorption and partial degradation within the host’s gastrointestinal (GI) tract. These genetically engineered probiotics are designed to maintain stability within the host’s digestive system and potentially exert their beneficial effects by surviving the digestive process in the GI tract. Apart from ensuring viability upon reaching the intestines, genetically engineered probiotics may also colonize and proliferate in the gut. Therefore, recombinant probiotics aim to address inherent limitations of probiotics, including reduced functionality in the host due to the presence of various enzymes in humans which can impede their effectiveness. As previously mentioned, probiotics may undergo distortion in the gut or fail to be assimilated by the host’s gut microbiota. Thus, genetically engineered microbes may achieve the initial aim of the use of probiotics, i.e., the increased abundance of microbes with advantageous potential for the host [93,94,95,96]. Notably, the development of genetically engineered probiotics is in its very beginning stages. For example, Moens et al. have reported on a four-strain genetically engineered probiotics mixture that achieved an increased production of butyrate, one of the SCFAs, in an in vitro model [96]. Large-scale studies are urgently needed in the near future in order to evaluate the efficacy and safety of genetically engineered microbes, especially in the clinical setting [97,98,99].

### 2.2. Phages in NAFLD/MASLD

Bacteriophages or phages are viruses that infect and kill bacteria. In addition to their ubiquitous presence, bacteriophages have been synthesized in laboratories since the early 1900s as a method of combating pathogenic bacteria. However, the subsequent development of antibiotics resulted in decreased interest in further phage research. Nevertheless, with the widespread emergence of multidrug-resistant pathogens (MDR) such as MDR *Acinetobacter baumanni* and *Pseudomonas aeruginosa* posing significant health challenges, there is a renewed focus on phage research. Recently, due to the alarming rise in the prevalence of NAFLD/MASLD, scientists are reconsidering the potential role of bacteriophages in treating this condition [100]. Gan et al. have shown that highly alcohol-producing *Klebsiella pneumoniae* may be a significant contributor to MASLD development. In their experimental model of highly alcohol-producing *Klebsiella pneumoniae* causing NAFLD, they used a phage specific to this bacterium that proved to decrease liver dysfunction and ameliorate cytokine expression. Gan et al. concluded that phage therapy could alleviate NAFLD [101]. However, no experiments have been performed in humans regarding phage therapy in NAFLD/MASLD. Therefore, further research on this topic could shed light on the utility of phage therapy among patients with NAFLD/MASLD. However, it is essential to highlight that safety concerns surrounding phage therapy, along with the technological and cost-related requirements, continue to be significant considerations. Additionally, beyond the safety issues of phage therapy, particularly concerning adverse effects, manipulating the gut microbiota through phages could present challenges due to potential non-selectivity regarding their targets. This issue should not be underestimated, as it could adversely affect the human gut microbiota [102].

### 2.3. Prebiotics and NAFLD/MASLD

According to the International Scientific Association for Probiotics and Prebiotics (ISAPP), prebiotics are “substrates selectively utilized by host microorganisms conferring a health benefit” [103]. Contrary to probiotics, which are live microorganisms, prebiotics are non-viable compounds, mainly—but non-exclusively–carbohydrates, that may have beneficial effects to the host. Although, before, only non-digestible carbohydrates were included in the definition of prebiotics, the latest ISAPP definition, which was published in 2017, clarifies that prebiotics could be non-carbohydrates as well [103]. In general, prebiotics work by promoting the growth of beneficial microbes in the gut. This is accomplished by the degradation of prebiotics by bacterial enzymes in the gut, which leads to an increased production of SCFAS. SCFAs are well known for their anti-inflammatory properties as well as their beneficial effects on intestinal barrier integrity. For example, oligosaccharides, such as fructans and galactans, have been shown to be related to an increased production of SCFAs, while also enhancing the abundance of *Bifidobacterium* in the host gut [104]. Through modulation of the gut microbiota, prebiotics alter the concentrations of SCFAs, BAs, and LPS transported to the liver via the enterohepatic circulation, thereby alleviating liver steatosis and NAFLD/MASLD [104,105]. Inulin and oligofructosaccharide (OFS) may decrease liver triglyceride levels and de novo lipogenesis in animal models of NAFLD [106,107,108,109,110,111,112,113,114,115,116]. Furthermore, in a small clinical study enrolling 14 patients with NAFLD/MASLD and NAS > 5, the administration of OFS for 9 months has been associated with a reduction in liver steatosis as proved by liver biopsy. In addition, an increase in the number of *Bifidobacterium* was noted in the same study. Polysaccharides are divided into storage polysaccharides, such as starch, and cell wall polysaccharides, such as non-starch polysaccharides. The latter not only faces limited absorption within the host gastrointestinal tract but may also exhibit anti-inflammatory and antioxidant properties [106,107,108,109,110,111,112,113,114,115,116]. According to the new ISAPP definition, apart from carbohydrates, other compounds, such as cocoa-derived flavonoids, are also considered to be prebiotics. These non-carbohydrate prebiotics may be found in chicory roots, almonds, garlic, chia seeds, artichokes, and other sources. In the era of the new definition of prebiotics, more studies are eagerly anticipated regarding the effects of prebiotics in NAFLD/MASLD.

### 2.4. Synbiotics and NAFLD/MASLD

Synbiotics are defined as the combination of probiotics and prebiotics [117,118,119,120]. This combination, particularly utilizing pro-anthocyanidins as prebiotics alongside a variety of probiotics, appears very promising. Pro-anthocyanidins combined with probiotics in a symbiotic form have been shown to be more effective in reducing de novo lipogenesis and stimulating fatty acid beta-oxidation [117,118,119,120]. Another study, using inulin and probiotics, specifically *Streptococcus Bifidobacterium* and *Streptococcus thermophilus*, as a symbiotic, has been demonstrated to improve NAFLD via its anti-inflammatory, antioxidant, and hypolipidemic properties [118]. An umbrella review of meta-analyses has recently reported an amelioration in serum CRP and TNF-a levels among patients with NAFLD/MASLD who were supplemented with symbiotics/probiotics [117]. However, only recently, Bilson et al. have reported that markers of fibrogenesis among 62 patients with NAFLD/MASLD and liver fibrosis ≳ 2 (at least F2) are not improved after supplementation with synbiotics [120]. Given the inconclusive nature of current research findings, it is imperative to undertake large-scale studies investigating the relationship between symbiotics and MASLD.

### 2.5. Postbiotics and NAFLD/MASLD

Postbiotics are defined according to the definition of ISAPP in 2021 as “a preparation of inanimate microorganisms and/or their components that confers a health benefit on the host” [121]. This definition refers to the existence of non-viable microbes, with or without their metabolites, which are beneficial for human health [122,123]. A paradigm is “yogurt based product for ambient distribution”. As such, conventional yogurt has been thermally treated to inactivate its starter cultures. Another paradigm is the infant formula for milk, which contains postbiotics, most of which are derivatives from *Bifidobacterium* or *Lactobacillus* [124,125]. Chelakkot et al. have studied the effects of *A. muciniphila* extracellular vesicles (EVs) on three major TJ proteins in mice fed a HFD and administered these EVs [126]. They have demonstrated that the expression of occludin, zonula occludens, and claudin-5 wasincreased in the HFD mice gavaged by A. muciniphila-derived EVs [126]. In the past, SCFAs such as butyrate, propionate, and acetate were considered the most well-known postbiotics, according to the previous definition of postbiotics. SCFAs are suggested to exhibit beneficial properties regarding the gut microbiota [11,12]. Butyrate, which is the most extensively studied SCFA until today, has been demonstrated to inhibit pro-inflammatory cells, such as M1 macrophages and neutrophils, while stimulating the anti-inflammatory M2 macrophages and T regulatory (Treg) cells. Apart from being a key player in immune cell regulation, butyrate enhances the production of mucin by goblet cells and the release of antimicrobial peptides from IECs [127]. Nevertheless, under the latest definition, the effectiveness of novel non-viable microbes, whether alone or in combination with their metabolites, will undergo further evaluation in forthcoming studies.

### 2.6. Fecal Microbiota Transplantation (FMT)

The efficacy of FMT has been documented in recurrent forms of *Clostridioides difficile* infection. More specifically, it is recommended to consider FMT therapy when encountering a fourth episode of *Clostridioides difficile* infection, provided that all prior episodes have been appropriately managed following the International Guidelines and that FMT is a feasible option [128,129]. However, FMT has been less rigorously studied in other medical conditions, such as NAFLD/MASLD. Notably, Xue et al. [130] performed a randomized clinical trial among 75 patients with NAFLD/MASLD. Xue et al. categorized the patients into two groups: one group received FMT, while the other, instead of FMT, received oral probiotics. Patients received FMT from healthy donors (allogenic and not autologous) via colonoscopy and then via three enemas for three subsequent days. After one month, the patients returned for re-examination. Then, the patients who underwent FMT showed decreased fat accumulation in their livers and reductions in serum lipid levels, which was achieved through the restoration of gut dysbiosis. Xue et al. confirmed the restoration of gut dysbiosis by performing 16 S rRNA sequencing of fecal samples. Moreover, they observed that lean patients with NAFLD/MASLD responded better to FMT, compared to obese patients with NAFLD/MASLD [130]. Interestingly, obese patients with MASLD have various beneficial interventions available to explore, including adopting healthy dietary strategies, engaging in regular exercise, and considering a prescribed regimen of weight loss medications. On the contrary, lean patients with NAFLD have very limited therapeutic options. In the era of resmetiron, which gained FDA approval for patients with NASH/MASH and moderate to severe fibrosis (at least F2) on 14th March 2024, FMT might be an alternative. Nevertheless, many more studies are needed to confirm or refute the true efficacy of FMT, even among this subgroup of lean patients with NASH/MASH and at least F2 fibrosis. In another study by Stols-Goncalves et al., 21 patients with NAFLD/MASLD underwent FMT as follows: 11 patients received autologous and 10 received vegan allogenic FMT. By using a multi-omics approach, Stols-Golcalves et al. confirmed alterations in the gut microbiome as well as changes in liver DNA methylation [131]. Despite the fact that FMT among patients with NAFLD/MASLD seems to be promising, there are still important issues to be resolved. Due to two cases of invasive infections involving extended-spectrum beta-lactamase (ESBL) *Escherichia coli* in immunocompromised patients, safety concerns persist as a significant issue. Therefore, according to the European Consensus and the FDA, the inclusion and exclusion criteria should be stricter to avoid any adverse effects [132,133]. In addition to carefully selecting donors, there is a suggestion to consider “super donors” or “keystone species’ donors.” This approach involves initially assessing the fecal microbiome of potential donors through 16S rRNA sequencing. Subsequently, donors with the desired “keystone species” are identified for potential use in FMT procedures [132,133].

## 3. Conclusions

Undoubtedly, the gut microbiome is pivotal in the pathogenesis of NAFLD/MASLD. Despite its significant contribution, there are numerous reservations about the efficacy of using probiotics, prebiotics, synbiotics, postbiotics, or FMT to treat NAFLD/MASLD. Furthermore, beyond uncertainties regarding their efficacy, the approaches employed to modulate the gut microbiota raise several safety concerns, notably regarding FMT. Nevertheless, particularly among lean patients with NAFLD/MASLD, who face limited treatment options, interventions such as supplementation with biotics or FMT to counteract gut dysbiosis could hold promise. Further large-scale studies are required to elucidate the efficacy and safety of the aforementioned therapeutic approaches.

## Figures and Tables

**Table 1:** Depicts main studies in animal models of NAFLD regarding the administration of probiotics and the subsequent results during the past five years.

Author/Year	Animal Model	Results	Remarks
Zhao et al., 2019 [57]	FGF-21 KO mice and C57BL/6 WT mice were fed a high fructose diet to induce NAFLD. They were administered *Lactobacillus rhamnosus* GG.	Improvement in serum adiponectin levels as well as enhancement of FGF-21 were noted.	Administration of *Lactobacillus rhamnosus* GGresulted in a reduction in serum adiponectin levels by promoting increases in FGF-21.
Wang et al., 2020 [58]	C57BL/6J mice were fed a high fat high cholesterol diet HFHCD. They were administered three strains of *Bifidobacterium adolescentis* and three strains of *Lactobacillus rhamnosus.*	Amelioration in gut microbiota composition was noted. Increased production of SCFAs was reported as well. Reductions in liver fat accumulation and inflammation were also observed.	Administration of three strains of *Lactobacillus rhamnosus* and three strains of *Bifidobacterium adolescentis* resulted in increased production of SCFAs as well as decreased F/B ratio in the gut.
Azarang et al., 2020 [59]	42 Male Sprague-Dawley rats were fed a high fructose diet. They were administered *Lactobacillus*, *L. acidophilus*, *L. casei*, *L. reuteri*, and *Bacillus coagulans*.	Reduction in serum triglycerides was reported. Reduction in hepatic triglyceride accumulation was noted.	Administration of the following probiotics solely or as a mixture: *Lactobacillus*, *L. acidophilus*, *L. casei*, *L. reuteri*, and *Bacillus coagulans* resulted in amelioration in oxidative stress markers.
Naudin et al., 2020 [60]	Female C57BL/6 mice were fed a high fat high carbohydrate Western diet. They were administered *Lactococcus lactis subsp cremoris* ATCC 19257.	Improvement in liver fat and inflammation was noted.	Administration of *Lactococcus lactis subsp cremoris* ATCC 19257 resulted in decreases in liver inflammation and hepatic fat accumulation.
Zhao et al., 2020 [61]	Insulin resistant C57BL/6J HFHF diet mice NAFLD model. They were administered *Lactobacillus plantarum* NA136.	Improvement in NAFLD-related inflammation. Increased abundance of beneficial bacteria in the gut microbiota.	Administration of *L.plantarum* NA136resulted in improvement of intestinal integrity and positive alterations in the gut microbiota composition.
Mu et al., 2020 [62]	C57BL/6J mice were fed a HFD/F to induce NAFLD. They were administered *Lactobacillus fermentum* CQPC06	Decreases in serum ALT, AST and triglyceride levels. Reductions in liver weight. Increases in the expression of ZO-1, occludin and claudin-1 in the gut.	Administration of *L. fermentum* CQPC06resulted in increased expression of tight junction proteins as well as increased in *Akkermansia* spp. and a decreased F/B ratio in the gut.
Lee et al., 2021 [63]	Male C57BL/6J mice were fed Western diet to induce obesity. They were administered *L. acidophilus*, *L. fermentum*, *L. paracasei*, and *L. plantarum.*	Reduction of the ratio of liver/body weight was noted. Reduction in pro-inflammatory cytokines, such as IL-6 and TNF-a was recorded as well.	Administration of *L. acidophilus*, *L. fermentum* and *L. plantarum* resulted in improvement of hepatic steatosis.
Zhang et al., 2021 [64]	Male C57BL/6 mice were fed a HFD. They were administered *Lactobacillus casei* YRL577and *L. paracasei* X11	*L. casei* YRL577 and *L. paracasei* X11exhibited higher BSH activity. *L. casei* decreased liver weight and proinflammatory cytokines as well.	*L. casei* YRL577 ameliorated liver steatosis by increasing the expression of FXR and FGF-15.
Yu et al., 2021 [65]	Male C57BL/6J mice were fed a Western diet to induce obesity. They were administered *Lactobacillus lactis* and *Lactobacillus pentosaceous.*	Improvement in serum liver enzymes. Improvement in tryptophan metabolism and the production of SCFAs.	Administration of *Lactobacillus lactis* and *Lactobacillus pentosaceous* resulted in improvement in tryptophan metabolism, which accounted for amelioration in NAFLD parameters.
Hong et al., 2021 [66]	Male C57BL/6J mice were fed a HFD. They were administered *Desulfovibrio vulgaris.*	Reduction in the expression of liver FAS. Increases in production of acetic acid.	Administration of *Desulfovibrio vulgaris* resulted in amelioration of liver steatosis via improving lipids metabolism in the liver and increasing the production of acetic acid.
Do et al., 2022 [67]	36 Male C57BL/6 mice were fed a HFD to induce They were administered *Bifidobacterium animalis* ssp. *lactis* MG741 (MG741).	Improvement in FAS and CREBP-1 expression. Reductions in liver steatosis score. Increased expression of tight junction proteins.	Administration of *Bifidobacterium animalis* ssp. *lactis* MG741 (MG741) resulted in increased expression of ZO-1 and occludin as well as decreased proinflammatory cytokines.
Hu et al., 2022 [68]	Male C57BL/6J mice were fed a HFD to induce NAFLD. They were administered 12 *Faecalibacterium prausnitzii* strains.	*F. prausnitzii* LC49 and LB8 improved fatty acid metabolism. In addition, it activated glutathione and tryptophan metabolism as well.	Administration of two strains of *F. prausnitzii* LC49 and LB8 resulted in modulations in the gut microbiota and increased production of SCFAs in the gut, apart from improved metabolism of critical substances.
Werlinger et al., 2022 [69]	60 Male C57BL/6 mice were fed a HFD to induce NASH. They were administered *Lactobacillus reuteri* MJM60668.	Increases in serum adiponectin levels. Decreases in serum ALT, AST and triglyceride levels. Reduction in the expression of FAS and SREBP genes. Increases in PPAR-a gene expression.	Administration of *Lactobacillus reuteri* MJM60668 resulted in reductions in liver weight, reduction in biomarkers of liver function as well as alterations in the gut microbiota, such as an increase in *Akkermansia* spp.
Riezu-Boj et al., 2022 [70]	18 Male C57BL/6 mice were fed a high fat high fructose (HFHF) diet. They were administered *Lactiplantibacillus plantarum* strain *DSM20174* (*L.p. DSM20174*)	Reductions in the M1/M2 ratio in the adipose tissue, as assessed by the changes in the expression of genes in macrophages. Less genes PPAR-a and SREBP expression as well.	Administration of *Lactiplantibacillus plantarum* strain *DSM20174 (L.p. DSM20174*) resulted in alterations in the diversity and the composition of the gut microbiota.
Nguyen et al., 2022 [71]	Male were fed a HFD to induce NAFLD. They were administered *Lactobacillus sakei* MJM60958 (MJM60958)	Reduction in body and liver weight. Decreases in serum ALT, AST and triglyceride levels. Decreased expression of SREBP-1 and FAS were reported. Increases in serum adiponectin levels. Increased expression of PPAR-a.	Administration of *Lactobacillus sakei* MJM60958 (MJM60958) resulted in amelioration in gut microbiota composition, as confirmed by increases in Verrucomicrobia and decreases in Firmicutes. In addition, a reduction in the expression of genes associated with liver fat accumulation was reported.
Han et al., 2023 [72]	SPF Male C57BL/6 mice fed HFD (D12492) to induce NASH. They were administered *Akkermansia muciniphila*	Prevention of hepatic inflammation Reduced liver M1 Reduced liver γδT cells Reduced liver TLR2 expression	*Akkermansia muciniphila* administration reduced M1 proinflammatory macrophages, thus reducing hepatic inflammation. It also ameliorated gut barrier integrity.
Yang et al., 2023 [73]	SPF Male FXR knockout mice. They were administered Cholesterol lowering probiotics, *Lactobacillus rhamnosus* DM9054 and *Lactobacillus plantarum* 86066.	Improved serum triglycerides and serum cholesterol levels Improved serum IL-1β and TNF-a levels	CL probiotics administration ameliorated gut microbiota. Reduced *Firmicutes* (harmful) and increased beneficial bacteria (*Actinobacteriota*).
Nian et al., 2023 [74]	60 SPF (specific pathogen free) Male C57BL/6 mice fed HFD. They were administered *Akkermansia. muciniphila* and *Bifidobacterium bifidum.*	Increased activation of liver FXR Decreased expression of intestinal FXR Increased tight junctions expression	Administration of *Akkermansia. muciniphila* or/and *Bifidobacterium bifidum* resulted in decreased liver inflammation and improvement in gut integrity and composition.
Zhao et al., 2023 [75]	30 Male C57BL-6 ob/ob mice (mutation in the leptin gene, resulting in increased appetite and obesity). They were administered *Lactobacillus oris* isolated from 75 Hainah centenarians.	Improved serum lipids profile.	Improvement in serum lipids profile could be attributed to the enhancement in cholesterol conversion to bile acids secretion. The potential role of the FXR-FGF-15 molecular pathway was pointed out.
Sun et al., 2023 [76]	Male ICR mice fed HFD. They were administered *Lactiplantibacillus plantarum* NKK20 strain (NKK20).	Improved serum lipid levels. Increased *Akkermansia muciniphila* abundance in the gut microflora.	NKK20 strain administration improved NAFLD, as confirmed by increased SCFAs production as well as amelioration in BAs profile.
Kim et al., 2023 [77]	CD-HFD Male C57BL-6 N mice. They were administered *Lactobacillus plantarum.*	Improvement in liver fat content. Increased serum L-arginine levels.	Administration of *Lactobacillus plantarum* increased serum L-arginine levels. This amelioration could account for improvement in NASH parameters.
Shin et al., 2023 [78]	96 Female C57BL/6 mice fed high- fructose-HFD to induce NASH. They were administered four *F. prausnitzii* strains (EB-FPDK3, EB-FPDK9, EB-FPDK11, and EB-FPYYK1)	Improvement in liver fat. Amelioration in hepatic fibrosis. Decrease in gut permeability.	Improvement in liver function parameters in NASH.
Lee et al., 2023 [79]	24 C57BL/6 Male mice fed HFD. They were administered *Limosilactobacillus fermentum* MG4294 and *Lactiplantibacillus plantarum* MG5289.	Improvement in liver triglyceride levels. Reduction in body weight. Decreases in serum ALT and AST levels.	Administration of *Limosilactobacillus fermentum* MG4294 and *Lactiplantibacillus plantarum* MG5289 resulted in improvement in hepatic lipid and proinflammatory parameters in NAFLD.
Kim et al., 2023 [80]	Male C57BL/6 mice were fed a NAFLD-induced diet (40 Kcal % fat, palm oil, 20 Kcal % frucrose and 2% cholesterol). They were administered *Lactiplantibacillus plantarum* LP158 (LP158), *Lactobacillus helveticus* HY7804 (HY7804), and *Lacticaseibacillus paracasei* LPC226 (LPC226) isolated from raw milk.	Improvement in serum lipids profile. Reduction in liver steatosis. Decrease in expression of lipogenesis genes, genes for inflammatory cytokines and fibrotic factors. Increased expression of genes promoting beta oxidation.	Administration of HY7804, LP158, and LPC226 resulted in improvement in NAFLD parameters.
Cao et al., 2023 [81]	24 C57BL/6 mice were administered a HFD. They were administered *Lactobacillus plantarum* ZJUIDS14.	Amelioration in liver steatosis. Increased expression of PPAR-a. Improved mitochondrial function, as confirmed by OXPHOS.	Administration of *Lactobacillus plantarum* ZJUIDS14 resulted in improvement in NAFLD due to mitigation of liver steatosis and gut permeability.
Li et al., 2024 [82]	Male C57BL/6J mice were fed western diet/carbon tetrachloride/dimethylnitrosamine (WD/CCl4/DEN)-induced model. They were administered *Bacteroides thetaiotaomicron.*	Reduction of body weight and liver fat accumulation. Restoration of gut microbiota composition. Improvement in liver lipids metabolism.	Administration of *Bacteroides thetaiotaomicron* resulted in amelioration in the gut microbiota composition, as manifested by reduction in F/B ratio as well as improvement in lipids metabolism in the liver.
Lee et al., 2024 [83]	Male C57BL/6 mice fed with HFD to induce NAFLD. They were administered *Lactiplantibacillus plantarum* DSR330 (DSR330).	Reduction in serum ALT, AST, ALP and serum triglyceride levels. Decreases in body weight. Increase in serum adiponectin levels.	Administration of *Lactiplantibacillus plantarum* DSR330 (DSR330) resulted in alleviation in hepatic steatosis by alterations in several molecular pathways.

Abbreviations: ALP: alkaline phosphatase; ALT: alanine-transferase; AST: aspartate transferase; BAs: bile acids; BSH: bile salt hydrolase; CD-HFD: choline deficient high fat diet; FAS: fatty acid synthase; F/B: *Firmicutes/Bacteroidetes*; FGF-15: fibroblast growth factor-15; FGF-21: fibroblast growth factor-21; FXR: farnesoid X receptor; HFD: high fat diet; HFHCD: high fat high cholesterol diet; HFHF: high fat high fructose diet; IL-1β: interleukin-1β; KO: knockout; NAFLD: nonalcoholic fatty liver disease; NASH: nonalcoholic steatohepatitis; OXPHOS: oxidative phosphorylation proteins; PPAR-a: peroxisome proliferator activated receptor alpha; SCFAs: short chain fatty acids; SREBP-1: sterol regulatory element binding protein-1; TNF-a: tumor necrosis factor-a; ZO-1: zonula occludens-1; WT: wild type.

## Abbreviations

ALT: Alanine Transferase; BAs: Bile Acids; BCFAs: Branched Chain Fatty Acids; BSEP: Bile Salt Export Pump; BSH: Bile Salt Hydrolase; ESBL: Extended Spectrum Beta Lactamases; EVs: Extracellular Vesicles; FMT: Fecal Microbiota Transplantation; FXR: Farnesoid X Receptor; GI: GastroIntestinal; GWAS: Genome Wide Association Studies; HOMA-IR: homeostasis model assessment insulin resistance; IECs: intestinal epithelial cells; IL-22: interleukin-22; IL-23: interleukin-23; ISAPP: International Scientific Association for Probiotics and Prebiotics; JAMs: junctional adhesion molecules; MASH: metabolic associated steatohepatitis; MASLD: metabolic dysfunction-associated steatotic liver disease; MS: metabolic syndrome; NAFLD: nonalcoholic fatty liver disease; NAS: NAFLD activity score; NASH: nonalcoholic steatohepatitis; NF-*k*B: nuclear factor Kappa b; NLRP3: nucleotide binding and oligomerization domain-like receptor protein 3; OSF: oligofructosaccharide; PAMPs: pathogen associated molecular patterns; PC: P-Cresol; SCFAs: short chain fatty acids; SNPs: single nucleotide polymorphisms; TJs: tight junctions; TLRs: Toll-like receptors; TMA: Trimethylamine; TMAO: Trimethyl-N-amine oxide; TNF-a: tumor necrosis factor-a; Tregs: T regulatory cells; WHO: World Health Organization.
